# The effect of the adversity quotient on student performance, student learning autonomy and student achievement in the COVID-19 pandemic era: evidence from Indonesia

**DOI:** 10.1016/j.heliyon.2021.e08510

**Published:** 2021-12-01

**Authors:** Asrop Safi'i, Imron Muttaqin, Sukino, Nur Hamzah, Chusnul Chotimah, Imam Junaris, Muh. Khoirul Rifa'i

**Affiliations:** a Fakultas Tarbiyah dan Ilmu Keguruan, Universitas Islam Negeri Sayyid Ali Rahmatullah, Tulungagung, Indonesia; b Fakultas Tarbiyah dan Ilmu Keguruan, Institut Agama Islam Negeri Pontianak, Indonesia; c Fakultas Tarbiyah dan Keguruan, Universitas Islam Negeri Sunan Ampel, Surabaya, Indonesia

**Keywords:** Adversity quotient, Achievement motivation, Learning autonomy, Student performance

## Abstract

This research investigates the effects of the adversity quotient introduced by Paul G. Stoltz on students achievement motivation, student learning autonomy and student performance. The study was conducted through an online survey with 218 participants from selected students of two Islamic senior high school in Indonesia. Data and information gathering from respondent analyzed by partial least square structural modelling using SmartPLS. This research revealed that adversity quotient were significant constructs affected on students achievement, students learning autonomy and student performance. This research opens a new paradigm for studying the adversity quotient and its implication for other educational aspects.

## 1 Introduction

An adversity quotient is a person's ability to manage difficulties and transform obstacles into opportunities. The adversity quotient is one factor that affects a person's success since it correlates positively with a person's performance. A person who has a high adversity quotient will also have high performance. People without adversity quotients will always depend on others, especially parents, peers, and others (Hurlock, 2000). They cannot take initiative and struggle greatly when confronting challenges. In the end, adversity quotients also has an impact on the possessor's performance, autonomy, and achievements. Success can be measured by one's ability to face difficulties in life (Stoltz, 2005), including flexibility, perseverance, and the ability to face problems in their duties and work. People who can solve problems more effeciently can control the situation and have a higher chance of success (Phoolka and Kaur, 2012).

Students require the adversity quotient in order to successfully face their problems and complete their duties and responsibilities in online learning. By having the adversity quotient, they can control the situation, take advantage of opportunities, and have a greater chance of success.

Several studies have revealed the effect of adversity quotients on workplace aspects. For instance, Sukardewi revealed a significant effect of adversity quotient on work ethics, school organization culture, and teacher performance (Sukardewi et al., 2013). Huijuan also reveals that the adversity quotient affects student performance (Huijuan, 2009). Other research suggests improving students' adversity quotient because it showed the affect impacted studies related to mathematics significantly (Suryadi and Santoso, 2017).

At the end of 2019 and early 2020, a new virus was found in Wuhan, China, namely, coronavirus disease 2019. This virus spread rapidly across the globe, causing the world to face a global pandemic. In the COVID-19 pandemic era, almost all sectors of the world's life were affected, including Indonesia. This condition impacted the daily life in many sectors of Indonesia.

The COVID-19 pandemic has affected the economy, education, personal and social life of Indonesian people. Some companies have closed due to the absence of buyers and the need to release too many employees. In the educational aspects, there was a change in learning patterns, and face-to-face meetings at schools and colleges which changed to online. It requires adaptation, readiness, and infrastructure support. Furthermore, it also requires the integration of all educational complements (Hermanto and Srimulyani, 2021). Teachers and students are required to provide energy and ability to online learning environments.

The COVID-19 pandemic has led to social restrictions and difficulty accessing educational facilities, so students can only obtain learning materials online or whenprovided by teachers by some other means. This condition requires adaptability to facing problems. So that adversity intelligence is needed for students. Preliminary research reveals that the adversity quotient affects students' adaptability (Rahayu, 2021), so students with high adversity quotients adapt quickly. It raises questions about the effect of adversity quotients on other student aspects.

Adversity quotients can help students carry out their duties and confront problems. Students need strength, fortitude, resilience, and intelligence to face difficulties effectively. Intelligence quotient (IQ) and emotional intelligence (EQ) are not enough to make a student successful; therefore, adversity quotients are necessary to manage obstacles and challenges.

This study requires senior high school students to answer research questions independently; older students will likely have the ability to understand the question and give proper answers. However, students in elementary schools and junior high schools will likely need assistance and guidance from their parents or others in online learning.

Additionally, the selected research location is the outstanding Islamic senior high schools, which at the time of this study had implemented online learning. For the study, two schools were eventually selected as the research locations due to possessing appropriate qualifications: Islamic Senior High School 1 (MAN 1) and Islamic Senior High School 2 (MAN 2) Pontainak West Kalimantan Indonesia.

## 2 Theory and hyphotheses development

### 2.1 Adversity quotient

The adversity quotient is a person's ability to face situations, problems, and obstacles in life. According to Stoltz, a person with an adversity quotient will be able to effectively face obstacles and take advantage of opportunities. The adversity quotient has four dimensions: control, ownership, reach, and endurance (Stoltz, 1997); it can be seen in a person's ability to maintain his or her composure when facing problems.

Previous studies have reported the effect of adversity quotients on various aspects of human life. For instance, the effect of adversity quotient on motivation, achievement (Ridho, 2016), learning outcomes dealing with mathematics (Rukmana et al., 2016), student entrepreneurial motivation (Wisesa and Indrawati, 2016), emotional maturity (Aryono et al., 2017), and student stress management (Jung, 2017).

### 2.2 Student performance

Performance assessment is a form of assessment that requires students to practice or apply knowledge obtained in various contexts according to the criteria for desired learning.

Research by Soysub and Jarinto and Huijuan reveals that the adversity quotient affects student performance (Huijuan, 2009) (Soysub and Jarinto, 2018). Mwivanda and Kingi also reveal that the adversity quotient is one dimension of student performance (Mwivanda and Kingi, 2019) and even suggest conducting AQ tests for teachers because of the importance of problems teacher's face in learning. Another previous study stated that people with high AQ perform more effectively and efficiently (Verma et al., 2017); there is a relationship between the level of adversity intelligence and the performance of a tutor (Solfema 2008), and the adversity quotient influences the performance of teachers. Thus, the adversity quotient offers a positive influence on performance (Rahmayanti et al., 2020). Therefore, this study hypothesizes a significant positive effect of adversity quotient intelligence on student performance.

H2 Adversity quotient will positively predict student performance.

### 2.3 Student learning autonomy

Brockett and Hiemstra said learning autonomy is an active learning activity derived from the encouragement of intention or motive to master a competency to overcome problems built with the provision of knowledge that already has (Brockett and Hiemstra, 1991). Learning autonomy is the ability to self-learn that can be expressed through an intensive process conducted by students to achieve the purpose of learning and mastery of lesson materials by using a variety of creative skills and techniques as well as the initiative of the student concerned; this ability can also be categorized as self-empowerment by students.

The ability to self-empower can be affected by the student adversity quotient (Kanjanakaroon, 2012). The ability to for students to successfully adjust to a new situation is necessary for autonomy learning to be effective; students who easily adjust to the learning environment will quickly develop positive learning attitudes. Among the things that can make students have the ability to adjust is the adversity quotient (Fitriany, 2008); this ability will impact autonomy when dealing with problems encountered. Based on the explanation above, the study hypothesizes that the adversity quotient directly affects student learning autonomy.

H3 Adversity quotient will positively predict student learning autonomy.

### 2.4 Student achievement motivation

Learning achievement is evidence of student success or their ability to successfully carry out learning activities. Understanding originates from learning interactions between teachers and students through development of student knowledge, attitudes, and skills. Students with a high adversity quotient (Nurhaidah, 2015; Nurhayati and Fajrianti, 2015) and student adaptability develop quickly and are more prone to achievement.

The research results also prove that the ability to survive and confront student problems also affects their achievement motivation (Suheil and Ratna Syifa'a, 2008). With the high motivation of learning, students will continue to learn to achieve the expected achievement (Ozen, 2017). Achievement is not obtained instantly, but with earnest efforts, students who can face learning difficulties will adjust quickly (Tian and Fan, 2014). This ability is predicted to affect student achievement. Several previous studies have revealed a relationship between adversity quotients and student achievement (Mz et al., 2017; Rukmana et al., 2016; Supardi, 2015; Suryadi and Santoso, 2017; Virlia, 2015).

These results show that students who can face problems and obstacles in learning also have significantly higher motivation. Suhel and Ratna support this statement by revealing the correlation between adversity quotient and achievement motivation (Suheil and Ratna Syifa'a, 2008). Therefore, we hypothesize that the adversity quotient affects student achievement motivation.

H1 Adversity quotient will positively predict student achievement motivation.

## 3 Method

This research was conducted from January 2021 to March 2021 through a survey conducted online. The data were obtained from student respondents at MAN 1 Pontianak and MAN 2 Pontianak. Respondents consisted of students of both males and females with an average age of 15–18 years. Students were selected based on the criteria of student achievement and autonomous learning in online instruction. The selection of these students was carried out by a teacher at the school.

The measurement of this research model was completed using SmartPLS 3.2 using the partial least squares structural equation modeling (PLS-SEM) procedure. The sample size is an important factor when used for partial least squares-SEM (PLS-SEM) with a sample size of at least 100 participants or meets a ratio between 5:1 and 10:1 (responses per item in the scale) to improve the confidence result (Goodhue et al., 2006). This research was approved by the Institute for Research and Community Service (Lembaga Penelitian dan Pengabdian Kepada Masyarakat) Pontianak State Institute for Islamic Studies (Institut Agama Islam Negeri) Pontianak, West Kalimantan Indonesia (protocol number B-137/In.15/LP2 M/PP.00.9/06/2021).

### 3.1 Instrumentation

The literature review is conducted as a guideline to determine definitions, concepts, and analysis related to the theoretical framework (Prasojo et al., 2020). A review of the literature was also used to determine the research instruments. This study uses a quantitative approach with four constructs: adversity quotient, student performance, student learning autonomy, and student achievement. The measurement of each variable uses the previous theory modified by researchers. Adversity quotient measurement uses Stoldz's opinion, which uses four dimensions: control, ownership, reach, and endurance (CORE) with a Likert scale consisting of strongly agree, agree, disagree and strongly disagree. This item was later developed and modified into six dimensions.

Student performance variables are measured using self-created indicators by opinion-based researchers (Glencoe, 2006) as follows: 1) obtain information, 2) process information, 3) assess the quality of information, 4) use information for a specific purpose, and 5) use information for presentation. Student learning autonomy is measured using five indicators put forward by Hiemstra: 1) setting learning objectives, 2) having learning skills, 3) having a scientific approach in learning, 4) having standards of success in learning and 5) having initiatives in learning (Brockett and Hiemstra, 1991). Finally, for student achievement motivation measured using opinions (McClelland, 1987) on the motivation of achievement, items are made by researchers with five indicators: 1) the need for achievement as measured by desire, 2) perseverance in achieving achievements, 3) the ability to utilize the help of others to achieve goals and careers, 4) have positive and negative feelings and personal responsibilities, and 5) be able to associate learning with a career.

### 3.2 Data collection

The instrument was distributed online using Google Forms. The data were obtained from two Islamic Senior High Schools (MAN 1 Pontianak and MAN 2 Pontianak). Data collection is done after obtaining permission from the school principals. The respondent was taken purposively with students who had minimum criteria. All respondents’ answers were entered in Excel, and the extension to CSV for SmartPLS input was changed. During the data collection process, all chosen respondents completed the Google Form. Two hundred eighteen respondents consisted of 79 male students and 139 female students.

## 4 Results

This study aims to determine the effect of adversity quotients on student performance, learning autonomy, and learning achievement. Previously formulated hypotheses are analyzed using SmartPLS 3. Construct is accepted as an explanation of the effect of adversity quotient on students performance, learning independence, and learning achievement.

### 4.1 Measurement models

**Figure 1:**
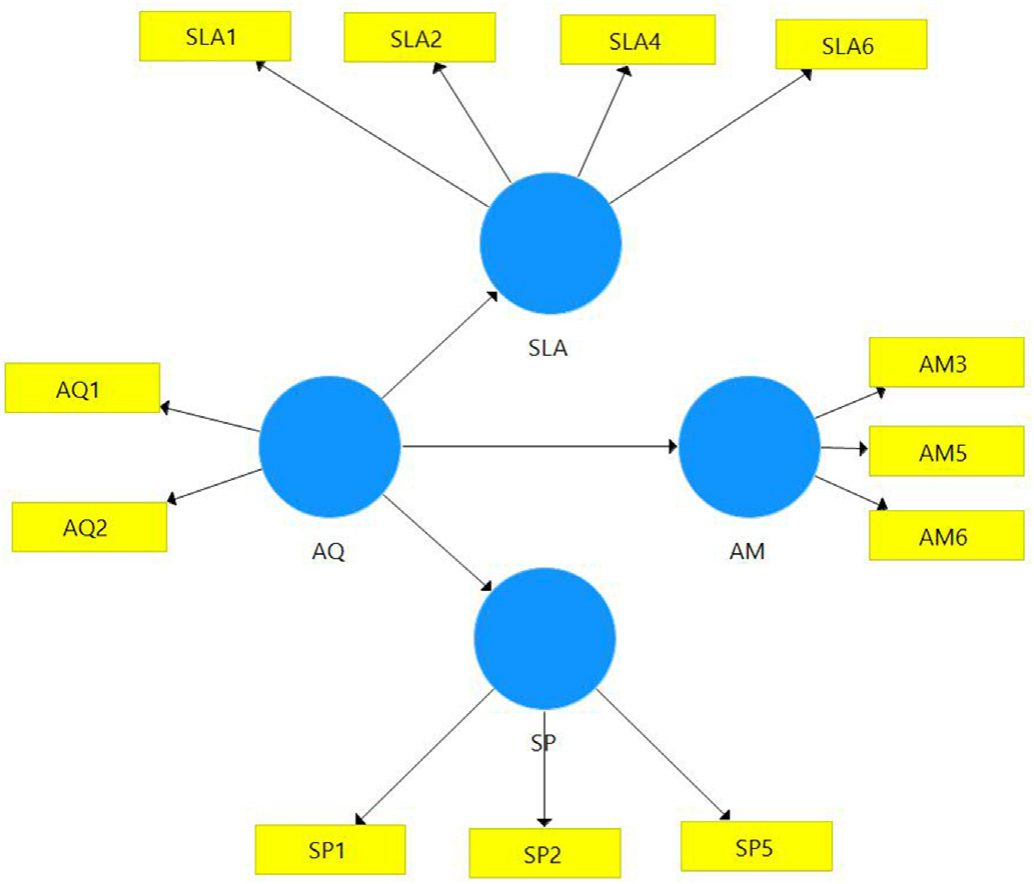
Proposed model.

Model measurements are performed by assessing the reliability and validity of the instrument. The indicator was assessed with three measurements: 1) indicator loading and internal consistency reliability, 2) convergent validity, and 3) discriminant validity (Hair et al., 2019). Figure 1.

### 4.2 Indicator loadings and internal consistency reliability

The results of the analysis using PLS-SEM were used to look at indicators in this study. Table 1 exhibits the detail of loadings. Three indicators from adversity quotient (AQ 3, AQ4 and AQ 5), three indicators from student achievement motivation (AM1, AM2, AM4), two indicators from students learning autonomy (SLA3, SLA5), and two indicators from student performance (SP3, SP4) were dropped since gained loading of below .708 (Hair et al., 2019). Internal consistency reliability should be reported through Cronbach's alpha (α) and composite reliability (CR). The values of α and CR in this study implemented the threshold set; α should be > .600 (Ghozali, 2011). CR should be >.708. Table 1 shows the details of both measure values.

**Table 1:** Reflective indicator loadings and internal consistency reliability.

	Item	Loading	α	CR	AVE
Adversity Quotient	AQ1	0,745	0,704	0,835	0,629
	AQ2	0,824			
	AQ6	0,808			
Students Achievement Motivation	AM3	0,810	0,751	0,856	0,664
	AM5	0,783			
	AM6	0,851			
Students Learning Autonomy	SLA1	0,775	0,671	0,819	0,603
	SLA2	0,824			
	SLA4	0,727			
Students Performance	SP1	0,781	0,663	0,816	0,597
	SP2	0,790			
	SP5	0,746			

### 4.3 Convergent validity

Convergent validity is associated with the validity of research instruments. Convergent validity intended to check the high-low relationship between indicators measures the same construct. This study uses SmartPLS to analyze instrument measurements. Convergent validity is met if the AVE value ≥.500 (Henseler et al., 2009). The instrument convergent validity analysis results showed that some indicators did not meet the convergent validity; some were removed because they did not meet the maximum AVE value limit. The remaining indicators met the convergent validity requirements (Table 2). Reliability tests are viewed based on Cronbach's alpha value. Based on the smartPLS output, the adversity quotient value is 0.534, students performance is 0.654, students learning autonomy is 0.603, and student achievement motivation is 0.608. Reliability is also seen from composite reliability. Variables that have a composite reliability value of >0.7 indicate high reliability. The results showed that the adversity quotient has a composite reliability of 0.811, student performance 0.786, student learning autonomy of 0.789, and student achievement motivation of 0.836 Table 1.

**Table 2:** Fornell-Larcker criterion.

	Adversity Quotient	Students Achievement Motivation	Students Learning Autonomy	Students Performance
Adversity Quotient	0,793			
Students Achievement Motivation	0,424	0,815		
Students Learning Autonomy	0,488	0,579	0,776	
Students Performance	0,485	0,540	0,526	0,772

### 4.4 Discriminant validity

Discriminant validity is the extent to which a construct is different from other constructs. By implementing the Fornell–Larcker criterion, the AVE scores of a construct should be lower than the shared variance for all model constructs. From the study results, the AVE scores of every construct are lower than that of its shared variance Table 2.

Therefore, discriminant validity was established based on the evaluation of the Fornell–Larcker criterion. Furthermore, discriminant validity can also be evaluated through the examination of cross-loadings. When a loading value on a construct is larger than those of all of its cross-loading values on the other constructs, discriminant validity emerges. Table 3 shows that all indicator values (bold) of the outer loading of every construct were above the values of all their cross-loadings on the other constructs. Thus, discriminant validity emerged from the cross-loading value examination. Discriminant validity problems also appear when HTMT values are higher than .900. The construct can be similar if HTMT shows a value of >.900 and lacks discriminant validity. Table 4 reported that all values of HTMT were lower than .900. The results indicate that the values significantly differed from 1.

**Table 3:** HTMT.

	AQ	AM	SLA	SP
Adversity quotient (AQ)				
Students achievement motivation (AM)	0,576			
Students learning Autonomy (SLA)	0,832	0,679		
Student performance (SP)	0,784	0,677	0,790

**Table 4:** Cross loading.

	Adversity Quotient	Student Achievement Motivation	Student Learning Autonomy	Student performance
AQ1	0,745	0,399	0,354	0,393
AQ2	0,824	0,365	0,495	0,426
AQ6	0,808	0,253	0,517	0,464
AM3	0,394	0,810	0,418	0,411
AM5	0,275	0,783	0,355	0,374
AM6	0,349	0,851	0,411	0,397
SLA1	0,480	0,400	0,775	0,345
SLA2	0,471	0,387	0,824	0,453
SLA4	0,390	0,346	0,727	0,435
SP1	0,456	0,450	0,448	0,781
SP2	0,381	0,353	0,420	0,790
SP5	0,407	0,311	0,346	0,746

Henseler, Ringle, and Sarstedt suggest a value for testing the validity of discriminant values not greater than 0.9 (Henseler et al., 2015). Table 3 indicates that all HTMT values are below 0.9, which suggests that all indicators based on the heterotrait-monotrait ratio are valid.

### 4.5 Structural model assessment

Structural model measurements use several steps. This measurement starts by calculating the reported collinearity with variance inflation factor (VIF) values. The relationship is determined with the test in the second stage, while the third stage is calculated coefficient determination (R^2^). In the fourth stage, f2 is calculated to determine the relevance of the construct; this calculation is intended to explain the selected endogenous construct. Regarding the R^2^ value and the effect size of f^2^ for the f^2^ value, the data are calculated using the blindfolding procedure to obtain the Q^2^, fifth, and sixth stage values. The data were also calculated using PLS-SEM through a blindfolding procedure in reporting Q^2^ values.

### 4.6 Collinearity issue

Furthermore, to test whether this model is worth using, a collinearity test is used. An instrument is eligible to proceed to the following process if the VIF value is less than 3 for the inner model, while for the outer model, it is smaller than 10. Adversity quotient is a predictor of student achievement motivation (VIF = 1,000), Adversity quotient is a predictor of student learning autonomy (VIF = 1,000), and the adversity quotient is a predictor of student performance (VIF = 1,000), Table 5.

**Table 5:** VIF values.

	AQ	AM	SLA	SP
Adversity quotient (AQ)				
Students' achievement motivation (AM)		1,000	1,000	1,000
Students learning Autonomy (SLA)				
Student performance (SP)				

### 4.7 Structural model relationship

**Figure 2:**
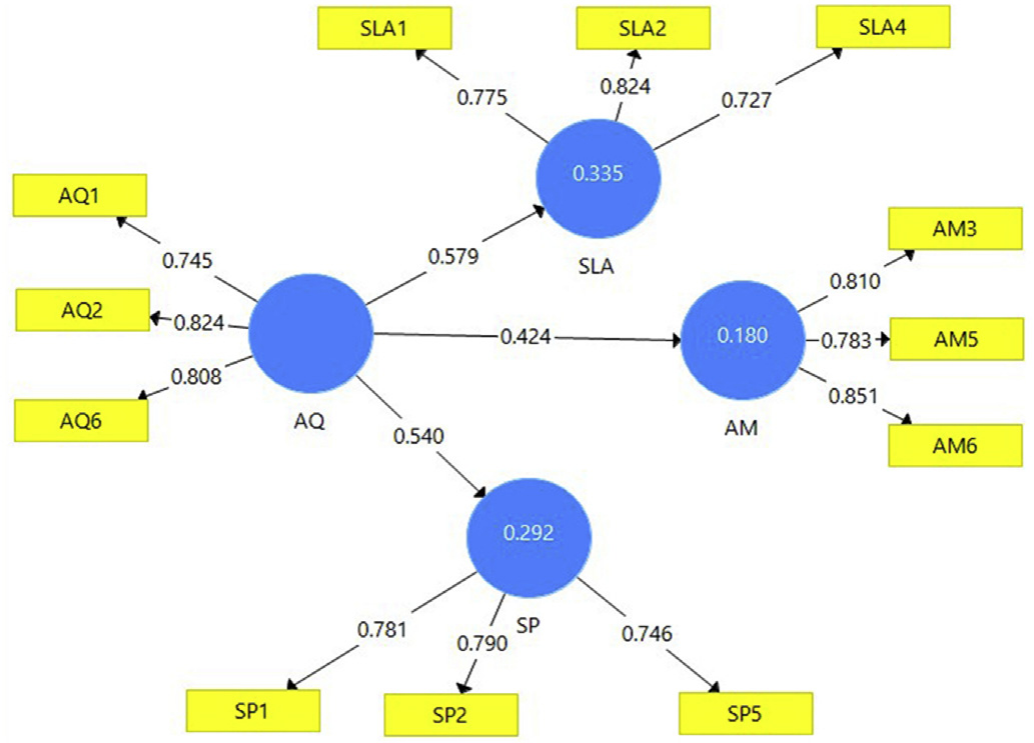
Final model.

Coefficient path calculations between endogenous and exogenous constructs was performed with 5,000 bootsrap subsamples (Figure 2). Applying 5% of significance (*one talled*). Adversity quotient was a significant predictor for student achievement motivation (β = 0,424; t = 7,284, p = 0,000), The adversity quotient was a significant predictor for student learning autonomy (β = 0,579; t = 12,570, p = 0,000), and the adversity quotient was a significant predictor for student performance (β = 0,540; t = 11.031, p = 0,000), Table 6.

**Table 6:** Final result.

	β	Mean	SD	T-Statistic	P Value	Sig
Adversity Quotient - > Students Achievement Motivation	0,424	0,433	0,058	7,284	0,000	Yes
Adversity Quotient - > Students Learning Autonomy	0,579	0,584	0,046	12,570	0,000	Yes
Adversity Quotient - > Students Performance	0,540	0,543	0,049	11,031	0,000	Yes

### 4.8 Coefficient of determination (R^2^)

The coefficient of determination (R^2^) is the variance proportion in endogenous variables predicted by exogenous variables. The range from 0 to 1; .75 is substantial, .50 is moderate, and .25 is considered weak (Chin, 1998). The *R*^*2*^ value of the Student Achievement motivation variable is 0.176 (weak), that of Student Learning Autonomy is 0.332 (weak), and that of Student Performance is 0.288 (weak). The detailed results of the R^2^ are shown in Table 7.

**Table 7:** Coefficient determination (R^2^).

	R^2^	R Square Adjusted	Consideration
Students Achievement Motivation	0,180	0,176	Weak
Students Learning Autonomy	0,335	0,332	Moderat
Students Performance	0,292	0,288	Weak

### 4.9 Effect size (f^2^)

Effect size (f2) measurement is taken by looking at changes in the coefficient of determination (R^2^) value to see how exogenous latent variables affect endogenous variables, whether or not they have a substantive effect (Ghozali, 2014). The f2 value .02 defines a small effect, .15 a medium effect and .35 means a large effect. Student learning autonomy gained the largest effect and student achievement motivation gained the smallest effect (.219). Detail F_2_ result reported on Table 8.

**Table 8:** *f*^2^ result.

	*f*^2^	Effect size
Students Achievement Motivation	0,219	Moderate
Students Learning Autonomy	0,504	High
Students Performance	0,412	High

### 4.10 Predictive relevance (Q^2^)

The Stone-Geisser test (*q*^*2*^) is a test that measures how well the observation value is generated by the model as well as its parameters. If the *q*^*2*^ value is greater than 0, then the model has predictive relevance, whereas if it is less than 0, it means that the model does not have predictive relevance (Ghozali, 2014). Steps to produce the Q^2^ values were conducted in PLS-SEM using the blindfolding procedure. If *q*^*2*^ is greater than 0, then the exogenous constructs have relevant predictors of endogenous constructs. Ghozali (2014) suggest that the predictive relevance values criteria .02 (*informs a small predictive*), 0.15 *informs a medium*, and 0.35 (*informs a large predictive*).

The blindfolding result shows that student learning autonomy has medium predictive relevance (Q^2^ ¼. 159), followed by student achievement motivation (Q^2^ ¼. 125), while student performance had the smallest predictive relevance of .109. Details for Q^2^ in this study are presented in Table 9.

**Table 9:** Predictive relevance.

	Q^2^	Predictive relevance
Students Achievement Motivation	0,125	Medium
Students Learning Autonomy	0,159	Medium
Students Performance	0,109	Medium

## 5 Discussion

This study aimed to investigate the effects of adversity quotients on students' achievement motivation, students' learning autonomy and students’ performance in the COVID-19 pandemic era. Through a bootstrapping process with 5,000 subsamples, the findings of this study revealed that adversity quotients exist to support student performance, learning autonomy and achievement during COVID-19.

Regarding the 1_st_ hypothesis, the t-statistic was 7.284, and the t value was below .05. The hypothesis was accepted because the t-statistic was greater than 1.96 with a .000 significance level. The *R*_*2*_ was .180, and the adjusted R squared was .176. This indicates that the adversity quotient has a weak effect on student performance. The blindfolding result was .125. This result indicates medium predictive relevance and indicates that exogenous constructs are relevant predictors of endogenous constructs. It can be inferred that adversity quotients affect students' achievement motivation. It can be inferred that adversity quotients affect students’ achievement motivation.

Adversity quotient affects students' achievement motivation through three indicators, namely, receiving advice that achievement is possible (AM3), the recognition of students that the learning materials taught support student achievement (AM5) and the students' thinking that learning materials in school are closely related to their achievement (AM6).

These findings are consistent with other studies on the COVID-19 pandemic reporting a correlation between adversity quotients and student achievement (Hariandayani and Nasution, 2021; Sugiarti et al., 2020; Susanti and Putra 2021; Ramadhani. 2021). Similar reports from previous research also confirmed this finding (Yodsakun, 2008; Zainuddin, 2011; Nurhayati and Fajrianti, 2015; Suryadi and Santoso, 2017). These researches revealed relations between adversity quotients and academic achievement.

Regarding the 2_nd_ hypothesis of the current study, three indicators that contributed are that students can set their own learning goals (SLA1), they have the necessary learning skills (SLA2), and they have standards (SLA4). Students can determine their own learning needs and goals. Adversity quotients also affect students' adaptability from offline to online learning, including the ability to access and use online learning to create their learning standards.

The output from SmartPLS provided a t-statistic of 12.570 and a t value below .05. The hypothesis was accepted because the t-statistic was greater than 1.96 with a .000 significance level. The *R*_*2*_ was .335, and the adjusted R squared was .332. This indicates that adversity quotients have weak effects on student performance. The blindfolding result was .159. This result indicates medium predictive relevance and indicates that exogenous constructs are relevant predictors of endogenous constructs. It can be inferred that adversity quotients affect student learning autonomy.

This study revealed that adversity quotients affect students' learning autonomy through three indicators (SLA1, SLA2 and SLA4). Adversity quotient affects students' ability to determine their own learning goals. This influence arises because of self-empowerment that is proven to be related to adversity intelligence (Kanjanakaroon 2012) and motivation. Students who are able to empower themselves will automatically be able to determine their own learning goals. Furthermore, students who can determine their own learning goals will also be able to choose learning skills that suit themselves and have their own standards. Therefore, adversity intelligence affects learning goals, necessary learning skills and student learning standards.

This finding is consistent with previous studies reporting that adversity quotients affect student learning autonomy (Patria and Silaen. 2020; Wahyuni, et, al.2020; Yazon and Ang-Manaig 2019). In normal times, adversity quotients have also been reported to affect student learning autonomy (Rahim, 2018). Student learning autonomy has been built through a long process since childhood age. Autonomy is an attitude that allows one to act freely, creatively, affect the environment, and have confidence and satisfaction without assistance from others (Masrun, 1986). Autonomy requires student responsibility, awareness, self-maturity, and self-discipline (Syam, 1999).

Regarding the last hypothesis, the SmartPLS output provided a t-statistic of 11.031 and a t value below .05. The hypothesis was accepted because the t-statistic was greater than 1.96 with a .000 significance level. The *R*_*2*_ was .292, and the adjusted R squared was .288. This indicates that the adversity quotient had a weak effect on student performance. Nevertheless, this study reveals the effect of adversity quotients on student performance.

Student performance in this study consists of finding, completing, collecting, and identifying materials obtained during online learning. In addition, the ability consists of explaining and constructing examples of material and writing and discussing the material. This finding is consistent with (Solfema, 2008; Kuhon, 2020; Verma et al., 2017; Rahmayanti et al., 2020) that reported a correlation between adversity quotients and performance and supported adversity quotients as psychological capital of student performance (Jafri, 2013). Although all the results indicate medium predictive relevance, all hypothesized variables reveal the effect of adversity quotients on all variables. Control and original ownership are the adversity quotient dimensions affecting students' achievement motivation, students' learning autonomy, and students' performance.

## 6 Conclusions

This study reveals the effect of adversity quotients on student performance, learning autonomy, and student achievement motivation. The three indicators of adversity quotients consist of students’ ability to control themselves when facing difficulties in learning (AQ1), the ability to know the causes of learning difficulties and being able to overcome (AQ2) and the ability to face problems in learning (AQ6).

These three indicators of adversity quotient affect students' ability to identify, find, collect and complete materials in learning (SP1); the ability to provide explanations and examples of learning materials (SP2); and the ability to talk, write and discuss materials obtained in learning (SP5). Three indicators of adversity intelligence affect students' learning autonomy during the COVID-19 pandemic despite weakness through student learning goals (SLA1), necessary learning skills (SLA2) and student learning standards (SLA4). This influence is caused by students’ motivation and empowerment because of their adversity intelligence. Three indicators of adversity quotients also affect students' ability to utilize advice and guidance to achieve achievement (AM3), confidence in the material taught by teachers is support for achievement (AM5), and students' belief that the material taught is related to their achievement (AM6).

This study reveals the influence of adversity quotients on three variables of the study. Although weak, this study managed to prove the influence. For further researchers, it is advisable to examine other factors that also affect three variables in addition to adversity intelligence. This study also has limitations. Namely, the number of respondents in the sample of this study was limited to 218 students. Other researchers can examine a larger sample or conduct research on college students. This study suggests that schools improve students’ adversity quotients.

## Declarations

### Author contribution statement

Asrop Safi’I, Imron Muttaqin and Chusnul Chotimah: Conceived and designed the experiments; Performed the experiments; Analyzed and interpreted the data; Wrote the paper.

Sukino, Nur Hamzah, Imam Junaris and Muh. Khoirul Rifa'i: Conceived and designed the experiments; Performed the experiments; Analyzed and interpreted the data.

### Funding statement

This research did not receive any specific grant from funding agencies in the public, commercial, or not-for-profit sectors.

### Data availability statement

Data included in article/supplementary material/referenced in article.

### Declaration of interests statement

The authors declare no conflict of interest.

### Additional information

No additional information is available for this paper.

## Figures and Tables

**Table:** 

Questions	
	Adversity Quotient
AQ1	I can control myself when faced with learning difficulties
	*Saya dapat mengendalikan diri saya ketika menghadapi kesulitan dalam belajar*
AQ2	I know the cause of my learning difficulties, but I can deal with them and know how to end them.
	*Saya mengetahui penyebab kesulitan belajar saya, tapi saya dapat dapat menghadapinya dan tahu cara mengakhirinya.*
AQ6	I know how to deal with problems in my learning
	*Saya tahu bagaimana menghadapi permasalahan dalam belajar saya*
	Student Learning Autonomy
SLA1	I can set my own learning goals *Saya dapat menentukan tujuan belajar saya sendiri*
SLA2	I have the learning skills I need
	*Saya mempunyai keterampilan belajar yang saya perlukan*
SLA4	I have a standard of success in my own learning
	*Saya memiliki standar keberhasilan dalam belajar saya sendiri*
	Achievement Motivation
AM3	When I get advice and guidance I do my best for my achievements.
	*Apabila saya mendapatkan nasehat dan bimbingan saya melaksanakan dengan sebaik-baiknya demi prestasi saya.*
AM5	The learning materialstaught by the teacher support my achievements. *Materi-materi pembelajaran yang diajarkan oleh guru menunjang prestasi saya.*
AM6	I think that the learning materials in school are closely related to my achievements.
	*Saya berpikiran bahwa materi pembelajaran di sekolah berkaitan erat dengan prestasi saya.*
SP1	I was able to find, complete, collect and identify the material I obtained from the learning process
	*Saya mampu menemukan, menyelesaikan, mengumpulkan dan mengidentifikasi materi yang saya dapatkan dari proses belajar*
SP2	I was able to give an explanation and make an example of the material I got in learning
	*Saya mampu memberikan penjelasan dan membuat contoh tentang materi yang saya dapatkan dalam belajar*
SP5	I can talk about, write and discuss the materials I have obtained during my studies.
	*Saya dapat membicarakan, menulis dan mendiskusikan materi yang sudah saya dapatkan selama belajar.*

## References

[bib1] Aryono S.Y., Machmuroch, Karyanta N.A. (2017). Hubungan antara adversity quotient dan Kematangan emosi dengan toleransi terhadap stres pada mahasiswa pecinta alam universitas sebelas maret. Jurnal Wacana.

[bib2] Brockett R.G., Hiemstra R. (1991).

[bib3] Chin W. (1998). The partial least squares approach to structural equation modeling. Mod. Meth. Busin. Res..

[bib4] Fitriany R. (2008).

[bib5] Ghozali I. (2011).

[bib6] Ghozali I., Kedua Edisi (2014).

[bib7] Glencoe McGraw-Hill (2006).

[bib8] Goodhue D., Lewis W., Thompson R. (2006). Proceedings of the 39th Annual Hawaii International Conference on System Sciences (HICSS'06).

[bib9] Hair J.F., Risher J.J., Sarstedt M., Ringle C.M. (2019). When to use and how to report the results of PLS-SEM. Eur. Bus. Rev..

[bib10] Henseler J., Ringle C.M., Sarstedt M. (2015). A new criterion for assessing discriminant validity in variance-based structural equation modeling. J. Acad. Market. Sci..

[bib11] Henseler J., Ringle C.M., Sinkovics R.R. (2009). New Challenges to International Marketing.

[bib12] Hermanto Y.B., Srimulyani V.A. (2021). The challenges of online learning during the covid-19 pandemic. Jurnal Pendidikan dan Pengajaran.

[bib13] Huijuan Z. (2009).

[bib14] Hurlock E.B. (2000).

[bib15] Hariandayani E., Nasution F.Z. (2021). Hubungan adversity quotient dengan motivasi berprestasi siswa SMA bani adam as medan. Jurnal Mahasiswa Fakultas Psikologi.

[bib16] Jung K.-S. (2017). The relation between adversity quotient and stress in university student. Kor. J. Youth Stud..

[bib17] Jafri M.H. (2013). A study of the relationship of psychological capital and students' performance. Busin. Persp. Res..

[bib18] Kanjanakaroon Jureeporn. (2012). Relationship between adversity quotient and self-empowerment of students in schools under the jurisdiction of the office of the basic education commission. Int. J. Learn..

[bib19] Kuhon F. (2020). A study on students adversity quotient and academic performance in English subject. J. Adv. Eng. Stud..

[bib20] Masrun (1986).

[bib21] McClelland D.C. (1987).

[bib22] Mwivanda M., Kingi P.M. (2019). Teachers’ adversity quotient dimension of control and students academic performance in secondary schools in Kenya. J. Educ. Train..

[bib23] Mz Z.A., Risnawati R., Kurniati A., Prahmana R.C.I. (2017). Adversity quotient in mathematics learning (quantitative study on students boarding school in pekanbaru). Int. J. Emerg. Mathem. Educ..

[bib24] Nurhaidah (2015). Pengaruh EQ dan AQ terhadap prestasi belajar ASKEB 1. Jurnal Ilmu Kebidanan Indonesia.

[bib25] Nurhayati N., Fajrianti N. (2015). Pengaruh adversity quotient (AQ) dan motivasi berprestasi terhadap prestasi belajar matematika. Form: Jurnal Ilmiah Pendidikan MIPA.

[bib26] Ozen S.O. (2017).

[bib27] Phoolka S., Kaur N. (2012). Adversity quotient: a new paradigm in management to explore. Int. J. Res. Soci. Sci. Manag..

[bib28] Prasojo L.D., Habibi A., Yaakob M.F.M., Pratama R., Yusof M.R., Mukminin A., Hanum F.J.H. (2020). Teachers’ burnout: a SEM analysis in an Asian context.

[bib29] Patria T.M., Silaen S.M.J. (2020). Hubungan Self Esteem dan Adversity Quotient dengan Kemandirian Belajar pada Siswa Kelas X di MAN 20 Jakarta Timur. IKRA-ITH HUMANIORA: Jurnal Sosial dan Humaniora.

[bib30] Rahayu A.P. (2021). Research result adversity quotient and self adaptation ability. Pendas Mahakam: Jurnal Pendidikan dan Pembelajaran Sekolah Dasar.

[bib31] Rahmayanti R., Egantara R.M.A.V., Ramadhan I. (2020). The influence of adversity quotient on the performance of honorary teachers in city of west bandung through motivation as intervening variables. Solid State Technol..

[bib32] Ridho E. (2016). Hubungan adversity quotient dengan motivasi berprestasi pada mahasiswa yang mengikuti organisasi intra (bemfa). Jurnal Penelitian UMM.

[bib33] Rukmana I., Hasbi M., Paloloang B. (2016). Hubungan adversity quotient dengan hasil belajar matematika siswa Kelas XI SMA negeri model terpadu madani palu. Jurnal Elektronik Pendidikan Matematika Tadulako.

[bib34] Ramadhani D. (2021). Hubungan Antara Adversity Quotient dan Motivasi Berprestasi pada Siswa yang Mengikuti SPP-SKS di SMPN 1 Sedati Sidoarjo. Experientia: Jurnal Psikologi Indonesia.

[bib35] Rahim A. (2018). Pengaruh Konsep diri dan adversity quotient terhadap Kemandirian santri. Fenomena.

[bib36] Soysub A., Jarinto K. (2018). The effects of multiple intelligent (IQ, EQ and AQ) on employee performance: a case of abc automotive. Co. Ltd. RMUTT Global Business Accounting and Finance Review (GBAFR).

[bib37] Stoltz P.G. (1997).

[bib38] Stoltz P.G. (2005).

[bib39] Suheil F., Ratna Syifa'a R. (2008). Adversity Quotient (AQ) dan motivasi berprestasi pada siswa program akselerasi dan program reguler. Gifted Review Jurnal Keberbakatan dan Kreativitas.

[bib40] Sukardewi N., Dantes N., Natajaya N. (2013). Kontribusi Adversity Quotient (AQ), etos kerja, dan budaya organisasi terhadap kinerja guru SMA negeri di Kota Amlapura. e-Journal Program Pascasarjana Universitas Pendidikan Ganesha.

[bib41] Supardi U.S. (2015).

[bib42] Suryadi B., Santoso T.I. (2017). Self-efficacy, adversity quotient, and students achievement in mathematics. Int. Educ. Stud..

[bib43] Solfema S. (2018). Adversity intelligence as a contributing factor of tutor’s performance. Eur. J. Educ. Stud..

[bib44] Syam N. (1999).

[bib45] Sugiarti R., Nurlaili A., Febriani U.F. (2020). Pengaruh adversity quotient terhadap motivasi berprestasi pada siswa cerdas istimewa. Philanthropy: J. Psychol..

[bib46] Susanti R., Putra G.P. (2021). Hubungan Adversity Quotient dengan Motivasi Berprestasi pada Siswa/i Kelas XII IPS II di SMAN 8 Batam Tahun 2018. Jurnal Ilmiah Zona Psikologi.

[bib47] Tian Y., Fan X. (2014). Adversity quotients, environmental variables and career adaptability in student nurses. J. Vocat. Behav..

[bib48] Virlia S. (2015). Hubungan adversity quotient dan prestasi belajar pada mahasiswa program studi psikologi universitas BM. Psibernetika.

[bib49] Verma S., Aggarwal A., Bansal H. (2017). The relationship between emotional intelligence (EQ) and adversity quotient (AQ). IOSR J. Bus. Manag..

[bib52] Wisesa D., Indrawati K.R. (2016). Hubungan adversity quotient dengan motivasi berwirausaha pada mahasiswa universitas udayana yang mengikuti program mahasiswa wirausaha. Jurnal Psikologi Udayana.

[bib53] Wahyuni U.T., Syahrilfuddin S., Putra Z.H. (2020). Hubungan adversity quotient dengan Kemandirian belajar matematika siswa Kelas IV sekolah dasar negeri 37 pekanbaru. Kontinu: Jurnal Penelitian Didaktik Matematika.

[bib54] Yazon A.D., Ang-Manaig K. (2019). Adversity Quotient®, emotional quotient and academic performance of Filipino student-parents. People: Int. J. Soc. Sci..

[bib55] Yodsakun A. (2008). Relationship between emotional intelligence (EQ) adversity quotient (AQ) and moral quotient (MQ) towards academic achievement of mattayom suksa two students. J. Educ..

[bib56] Zainuddin (2011). Pentingnya adversity quotient dalam meraih prestasi belajar. Jurnal Guru Membangun.

